# Changing Epidemiology of Hepatitis C Virus Genotype among Patients with Human Immunodeficiency Virus/Hepatitis C Virus Co-Infection in China

**DOI:** 10.1371/journal.pone.0161844

**Published:** 2016-09-07

**Authors:** Weilie Chen, Baolin Liao, Fengyu Hu, Jingmin Nie, Yun Lan, Huiqin Li, Ruichao Lu, Yanqing Gao, Yuxia Song, Qingxia Zhao, Yuhuang Zheng, Xiaoping Tang, Weiping Cai

**Affiliations:** 1 Department of Infectious Disease, Guangzhou No. 8 People’s Hospital, Guangzhou Medical University, Guangzhou, China; 2 Department of Infectious Disease, Yunnan Provincial Infectious Disease Hospital, Yunnan, China; 3 Department of Infectious Disease, Guangxi Zhuang Autonomous Region Longtan Hospital, Guangxi, China; 4 Department of Infectious Disease, Beijing Youan Hospital, Capital Medical University, Beijing, China; 5 Department of Infectious Disease, Xinjiang No. 6 People’s Hospital, Xinjiang, China; 6 Department of Infectious Disease, Zhenzhou No. 6 People’s Hospital, Zhenzhou, China; 7 Department of Infectious Disease, the Second Xiangya Hospital, Zhongnan University, Changsha, China; University of Cincinnati College of Medicine, UNITED STATES

## Abstract

**Background:**

Co-infection with hepatitis C virus (HCV) has become the most common cause of death in human immunodeficiency virus (HIV) infected patients on antiretroviral therapy. The distribution of HCV genotypes varies with geographical regions and time, and limited studies have focused on the HCV genotype in HIV/HCV co-infection.

**Methods:**

The distribution of HCV genotypes was evaluated in 414 patients with HIV/HCV co-infection in three regions (South, Central and Northwest) of China from 2008 to 2010. The NS5B region of HCV was characterized using nested reverse transcription polymerase chain reaction. Nucleotide sequences obtained were subjected to phylogenetic analysis, and genotypes were assigned using published reference genotypes.

**Results:**

Genotype 3 was the most prevalent HCV strain (36.2%), followed by genotype 6 (30.0%), genotype 1 (28.5%), genotype 2 (5.1%), and genotype 5 (0.2%). The distribution varied geographically. Genotype 6 (37.6%) was the predominant strain while genotype 1 (20.2%) was less common in the South compared to the Central and Northwest regions (all P < 0.001). The distribution also varied temporally. There was no significant difference in genotype distribution in Guangdong (a province in the South region), between patient cohorts from 2005–2008 and 2009–2010. However, outside Guangdong, genotypes 3 and 6a became significantly more prevalent (22.4% vs.42.2%, P< 0.001; 8.0% vs. 19.8%, P = 0.004), and genotype 1 less prevalent (54.4% vs.26.6%, P< 0.001) over time.

**Conclusion:**

The most dramatic shift in genotypic distribution was the movement of HCV genotypes 3 and 6a outside of Guangdong in HIV/HCV co-infected patients. This movement appeared closely associated with transmission via injected drug use.

## Introduction

Infection with hepatitis C virus (HCV) is a major cause of chronic liver disease worldwide. It is estimated that approximately 160 million individuals are chronically infected with HCV [1]. Additionally, about 40 million people have been infected by human immunodeficiency virus (HIV) globally. HIV and HCV share transmission routes including contaminated blood transfusion, sexual intercourse, and needle-sharing in injection drug users (IDU). Recognition of the burden of HIV/HCV co-infection is increasing, and is estimated to affect 5–7 million people worldwide [2]. Persistent HCV infection is associated with the development of liver cirrhosis, hepatocellular cancer and death [3]. HIV infection accelerates the natural progression of HCV infection, therefore HCV co-infection has become the most common cause of death in HIV/acquired immune deficiency syndrome (AIDS) patients on antiretroviral therapy [4].

Seven HCV genotypes (1 to 7) and a large number of subtypes have been identified and are distributed worldwide. Genotype 1 is estimated to be the most prevalent globally, with over one-third of patients located in East Asia. East Asia also accounts for the greatest number of genotypes 2 and 6, while North Africa and the Middle East have the largest number of genotype 4. The majority of genotype 5 patients are located in Southern and Eastern sub-Saharan Africa [5].The novel genotype 7 was identified in patients from Canada and Belgium, possibly infected in Central Africa [6].

The management and antiviral response to treatment are dependent in part on the HCV genotype, and the distribution of HCV genotypes may vary within the same geographic region over time. Therefore, the epidemiological description of HCV genotypes is critical to inform disease control, prevention and treatment strategies. Recent studies indicate that genotypes 1b and 2a remain the most prevalent HCV strains in China, while genotypes 3 and 6 are increasing in their geographic distribution [7]. However, there has been limited research focusing on HCV genotypes in HIV/HCV co-infection. Therefore, we investigated the distribution of HCV genotypes in HIV/HCV co-infected patients from different regions in China over time.

## Methods

### Patients

All patients were enrolled from the outpatient clinics or were hospitalized in one of seven centers between 2008 and 2010. The inclusion criteria were as follows: (1) HIV-1 positive, (2) older than 18 years of age, (3) positive HCV antibody (anti-HCV) and detectable HCV RNA > 1000 IU/mL. These patients were initially recruited as part of a large multicenter HCV treatment trial, and this study represents a secondary analysis of data from that trial at the time of enrollment. The exclusion criteria were as follows: (1) hepatitis B virus co-infection, (2) evidence of liver disease because of other etiology, (3) use of hepatotoxic drugs or regular consumption of alcohol, (4) received antiviral (HIV or HCV) therapy previously, and (5) individuals with decompensated cirrhosis, severe cytopenias, pregnancy, breast-feeding status, renal failure, heart failure, or an AIDS-defining illness. In total, 414 HIV/HCV co-infected patients were recruited. Three different geographical regions of China were represented: 317 (76.6%) patients from the South (Guangdong province, n = 97; Yunnan province, n = 127; Guangxi province, n = 78; Hunan province, n = 15), 60 (14.5%) patients from the Central (Shanxi province, n = 31; Hebei province, n = 4; Henan province, n = 20; Beijing, n = 5), and 37 (8.9%) patients from the Northwest (Xinjiang, n = 37). Blood samples were stored at -70°C prior to HCV genotyping. Demographic and clinical data such as age, gender and collection date were kept confidential. All samples were number-coded and anonymous. The study protocol was conducted within the guidelines of the 1975 Declaration of Helsinki and was approved by the ethics committee of Guangzhou No.8 People’s Hospital. Written informed consent was obtained from all patients. The trial registration number is ChiCTR-TRC-00000407 (http://www.chictr.org.cn/showproj.aspx?proj=9126).

### Serological and biochemical assays

The presence of HIV-1 antibody was determined by enzyme-linked immunosorbent assay (ELISA) (Wantai Biological, China). ELISA-positive samples were confirmed by the HIV-1/2 Western blot immune assay (MP Biomedicals, Singapore).The presence of anti-HCV antibody in blood samples was determined by ELISA (Zhongshan Biotechnology Co. Ltd., China). Serum alanine aminotransaferase (ALT) and aspirate aminotransferase (AST) levels were determined by commercial kits. The upper limit normal (ULN) of ALT and AST were 40 U/L for both male and female patients.

### Viral extraction, nested reverse transcription polymerase chain reaction (RT-PCR) amplification and sequencing

Viral HCV RNA was extracted from serum samples using a QIAamp Viral RNA Mini Kit (Qiagen, Germany) following the manufacturer’s protocol. The primers of NS5B region for nested RT-PCR, which were designed based on HCV H77 (GenBank Accession NO.AF009606), are described previously [8] with slight modification: out forward (OF) 5'-TGGGSTTYTCSTATGAYACCMGBTGYTTTGA-3' (nt 8245 ~ 8275), out reverse (OR) 5'-ARTACCTRGTCATAGCCTCCGTGAA-3' (nt 8640 ~ 8616), inner forward (IF) 5'-TATGAYACCCGCTGYTTTGACTCCAC-3' (nt 8256 ~ 8281), inner reverse (IR) 5'-GTCATAGCCTCCGTGAAGGCTC-3' (nt 8632 ~ 8611). First round PCR using primer OF and OR was conducted by the one-step RT-PCR kit (Qiagen, Germany) with the following conditions: 50°C for 30min, 95°C for 15 min, followed by 35 cycles of 94°C for 30 sec, 55°C for 40 sec and 72°C for 60 sec. Second round PCR using primer IF and IR was conducted with the following conditions: 94°C for 2 min, followed by 30 cycles of 94°C for 25 sec, 55°C for 35 sec and 72°C for 50 sec. A final extension step of 72°C for 10 min was performed in both rounds of PCR amplification. After purification with the QIAquick PCR Purification kit (Qiagen, Germany), samples were sequenced using the inner primers on an automated sequencer (ABI PRISM 3130 Genetic Analyzer, Applied Biosystems, USA).

### HCV genotyping and phylogenetic analyses

The nucleotide sequences obtained were edited with Sequence Scanner 1.0 (ABI, USA). HCV genotype was determined after alignment with reference sequences retrieved from HCV sequence database (http://hcv.lanl.gov) representing the major genotypes and subtypes of HCV. Genotypes of HCV isolates were assigned based on the phylogenetic analysis of NS5B sequences. Neighbor-joining trees were estimated by using Maximum Composite Likelihood Substitution Model implemented in MEGA 4.0. Reliability of the phylogenetic trees was assessed by 1000 bootstrap resampling. The reference sequences used in phylogenetic analysis were obtained from the Genbank: [1a] AB520610, EU781824, EU781769, FJ205868, M62321, M67463; [1b] D10934, L02836, M58335; [1c] D14853, AY051292, AY651061; [2a] D00944, AF238482, AF238483; [2b] AF238486, AB030907, D10988; [2c] D50409; [3a] AF046866, D17763, D28917; [3b] D49374; [4a] Y11604; [5a] Y13184; [6a] Y12083, AY859526, DQ480522; [6b] D84262; [6n] AY878652; [6u] EU408331; [6v] EU158186, EU798760, EU798761, FJ435090.

### Statistical analysis

Data was analyzed using the statistical package SPSS (version13.0; SPSS, Inc., Chicago, IL). Results were presented as a mean±SD or number(%) of patients. Chisquare was used for categorical variables. Mann-Whitney or Kruskal-Wallis test was used for similar comparison of nonparametric data. A two tailed p-value of <0.05 was considered statistically significant.

## Results

### Characteristics of the patients with HIV/HCV co-infection

The majority of patients enrolled for study were male (72.9%). The age and HCV RNA levels among patients from the three regions were statistically different (P < 0.001) (Table 1). Patients from the Central region were older on average than those from the South (P < 0.001) and Northwest regions (P < 0.001), while ages were similar between the South and Northwest regions. In terms of transmission risk behaviors, patients from the South region were more likely to be IDU and less likely to have received HCV via blood transfusion than those from the Central region (both P< 0.001). Risk factors in the majority of patients from the Northwest were unknown.

**Table 1:** Characteristics and genotypes of HCV in patients with HIV/HCV co-infection^a^.

	South	Central	Northwest	Total	P value
	N = 317	N = 60	N = 37	N = 414	
Age	36±6	42±8	34±7	37±7	<0.001
Sex (Male%)	240 (75.7)	31 (51.7)	31 (83.8)	302 (72.9)	<0.001
HCV viral load (log_10_IU/mL)	5.54±1.07	6.07±1.09	6.15±1.01	5.67±1.09	<0.001
ALT (U/L)	66±47	59±39	74±47	66±46	0.343
AST (U/L)	63±45	52±33	53±23	60±42	0.868
Risk behaviors (%)					<0.001
IDU	254 (80.1)	31 (51.7)	14 (37.8)	299 (72.2)	
Sex	48 (15.1)	3 (5.0)	2 (5.4)	53 (12.8)	
Blood transfusion	5 (1.6)	17 (28.3)	1 (2.7)	23 (5.6)	
Unknown	10 (3.2)	9 (15.0)	20 (54.1)	39 (9.4)	
Genotype					<0.001
Genotype 1 (%)	64 (20.2)	37 (61.7)	17 (45.9)	118 (28.5)	
1a	22 (6.9)	0 (0)	1 (2.7)	23 (5.6)	
1b	42 (13.2)	37 (61.7)	16 (43.2)	95 (22.9)	
Genotype 2 (%)	2 (0.6)	18 (30.0)	1 (2.7)	21 (5.1)	
2a	2 (0.6)	18 (30.0)	1 (2.7)	21 (5.1)	
Genotype 3 (%)	132 (41.6)	2 (3.3)	16 (43.2)	150 (36.2)	
3a	58 (18.3)	2 (3.3)	12 (32.4)	72 (17.4)	
3b	74 (23.3)	0 (0)	4 (10.8)	78 (18.8)	
Genotype 5 (%)	0 (0)	0 (0)	1 (2.7)	1 (0.2)	
5a	0 (0)	0 (0)	1 (2.7)	1 (0.2)	
Genotype 6 (%)	119 (37.6)	3 (5.1)	2 (5.4)	124 (30.0)	
6a	98 (30.9)	1 (1.7)	2 (5.4)	101 (24.4)	
6n	17 (5.4)	1 (1.7)	0 (0)	18 (4.3)	
6u	0 (0)	1 (1.7)	0 (0)	1 (0.2)	
6v	4 (1.3)	0 (0)	0 (0)	4 (1.0)	

IDU, injection drug users; ALT, alanine aminotransaferase; AST, aspirate aminotransferase

^a^Data are presented as a mean±SD or number (%) of patients.

**Fig 1:**
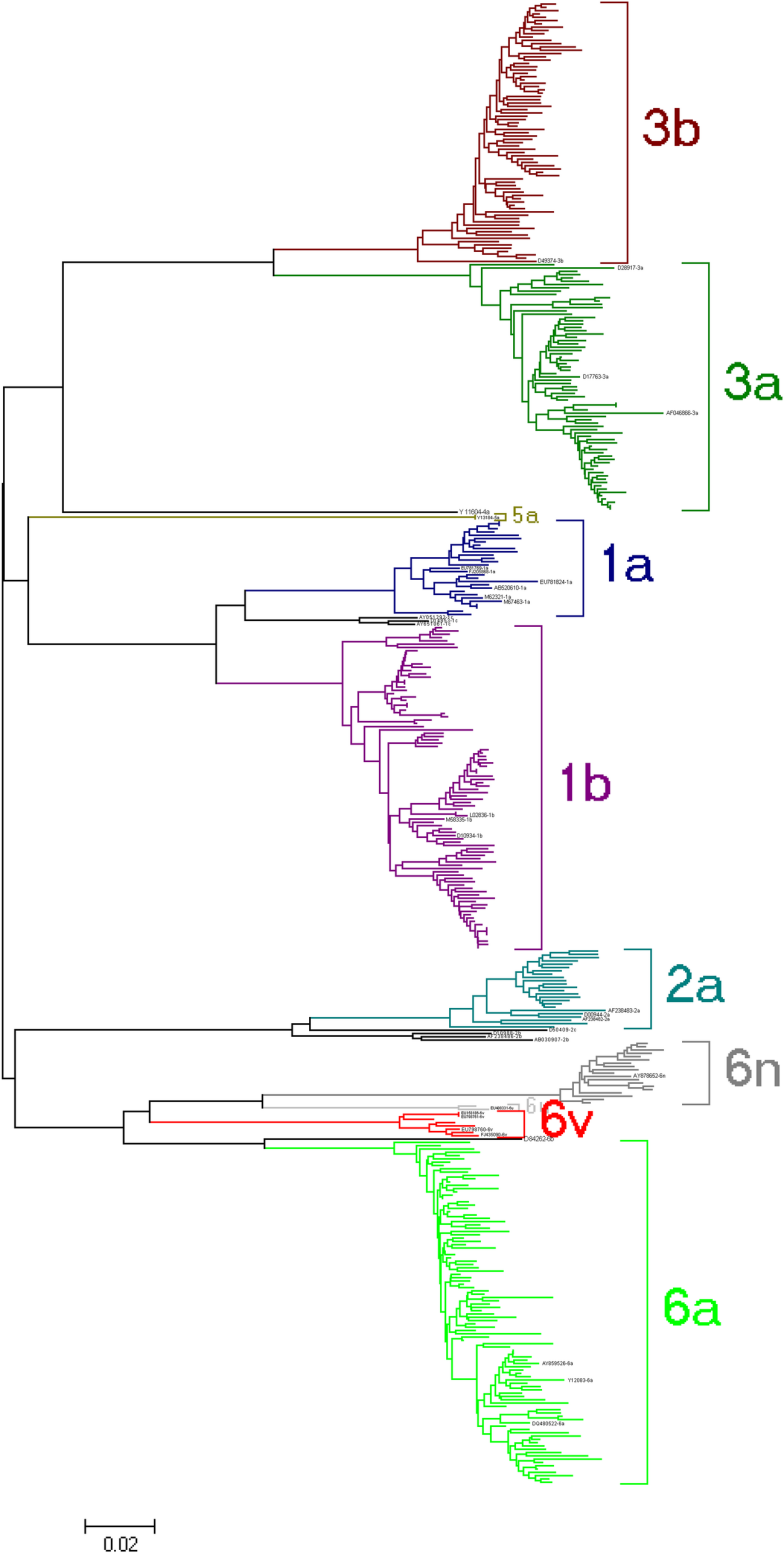
Phylogenetic trees of HCV NS5B gene of patients with HIV/HCV co-infection. There were 5 HCV genotypes and 10 subtypes identified in our studied population, but their frequencies were not equally distributed amongst the group.

There were 5 genotypes and 10 subtypes of HCV identified in our patient population (Fig 1). Age varied among patients with different HCV genotypes (P = 0.001), with the genotype 2 and 1 groups containing the oldest patient populations (Table 2). There were also significant differences in age among groups with different HCV subtypes (P < 0.001). Further analyses reveal the subtype 1b patient group was older than the subtype 6a (P < 0.001), 3a (P < 0.001) and 3b (P = 0.015) groups, respectively (Table 3).

**Table 2:** Age distribution among different HCV genotypes^a^.

	N	Mean age (SD)	Minimum age	Maximum age
Genotype 1	118	38±7	24	65
Genotype 2	21	43±8	27	59
Genotype 3	150	36±6	21	52
Genotype 5	1	37	37	37
Genotype 6	124	36±6	22	53
Total	414	37±7	21	65

^a^Data are presented as a mean±SD of patients.

**Table 3:** Age distribution among different HCV subtypes^a^.

HCV subtype	N	Mean age (SD)	Minimum age	Maximum age
1a	23	33±6	25	45
1b	95	39±7	24	65
2a	21	43±8	27	59
3a	72	35±6	21	52
3b	78	37±5	26	48
5a	1	37	37	37
6a	101	36±6	22	53
6n	18	36±5	28	44
6u	1	33	33	33
6v	4	38±2	37	41
Total	414	37±7	21	65

^a^Data are presented as a mean±SD of patients.

### Distribution of HCV genotypes in patients with HIV/HCV co-infection in China

The HCV genotypes identified in this study were distributed unequally. In decreasing order, the predominant HCV strain was genotype 3, followed by genotype 6, genotype 1, genotype 2, and genotype 5. The frequency of genotype 3, the predominant form, ranged from 3.3% to 43.2% in different regions. Genotype 6, the second most predominant form, demonstrated robust subtype distribution, with subtype 6a (24.4%) as the major form; however, other subtypes (6n, 6u and 6v) were also present. (Table 1).

The HCV genotype was unevenly distributed by region (Table 1). Distribution of genotype 3 differed significantly among the 3 regions (P < 0.001). The proportion of patients with genotype 3 identified in the Central region was significantly lower compared with the South and the Northwest regions (both P < 0.001) although no difference was observed between the South and Northwest groups. Genotype 6 was the predominant genotype in the South, but accounted for a small proportion of the populations in the Central and Northwest regions (both P < 0.001). Additionally, genotype 1 was infrequently in the South as compared to the Central (P < 0.001) and the Northwest regions (P < 0.001), but was present at similar frequencies in the Central and Northwest populations. Lastly, results indicate only a few patients were infected with subtype 2a, almost exclusively found in the Central region, and the only case of genotype 5 was located in the Northwest region.

### Association of HCV genotypes and risk behaviors

The distribution of HCV genotypes in HIV/HCV co-infected patients in China varied by geographic region. We hypothesize this variation in distribution may result from differences in the route of HCV transmission. We then examined the relationship between HCV genotypes and the risk factors for transmission and noted that the frequency of HCV genotypes differed based on route of transmission (Table 4). Patients from the South were mainly infected through IDU (80.1%), with a higher frequency of subtype 6a (33.5%), followed by subtype 3b (23.6%) and 3a (17.7%). Additionally, no patients with subtype 6a were infected via blood transfusion, which was the most common route for subtypes 1b and 2a (60%). Similarly, all patients infected through blood transfusion from the Central region had HCV subtypes 1b or 2a. Subtype 1b (74.2%) was mostly found in patients infected through IDU, followed by subtype2a (16.1%), while subtype 3a, 6a and 6n were also identified for this transmission group. Considering the small sample size and unknown transmission routes in more than half of patients from the Northwest region, we did not explore the relationship between genotype and risk behaviors in this group.

**Table 4:** Genotypes distribution in patients with different risk behaviors^a^.

		Sample	1a	1b	2a	3a	3b	5a	6a	6n	6u	6v
**South**	IDU	254	21 (8.3)	28 (11.0)	0 (0)	45 (17.7)	60 (23.6)	0 (0)	85 (33.5)	12 (4.7)	0 (0)	3 (1.2)
	Sex	48	1 (2.1)	10 (20.8)	1 (2.1)	10 (20.8)	10 (20.8)	0 (0)	12 (25.0)	4 (8.3)	0 (0)	0 (0)
	Blood transfusion	5	0 (0)	2 (40.0)	1 (20.0)	1 (20.0)	0 (0)	0 (0)	0 (0)	0 (0)	0 (0)	1 (20.0)
	Unknown	10	0 (0)	2 (20.0)	0 (0)	2 (20.0)	4 (40.0)	0 (0)	1 (10.0)	1 (10.0)	0 (0)	0 (0)
**Central**	IDU	31	0 (0)	23 (74.2)	5 (16.1)	1 (3.2)	0 (0)	0 (0)	1 (3.2)	1 (3.2)	0 (0)	0 (0)
	Sex	3	0 (0)	1 (33.3)	2 (66.6)	0 (0)	0 (0)	0 (0)	0 (0)	0 (0)	0 (0)	0 (0)
	Blood transfusion	17	0 (0)	8 (47.1)	9 (52.9)	0 (0)	0 (0)	0 (0)	0 (0)	0 (0)	0 (0)	0 (0)
	Unknown	9	0 (0)	5 (55.6)	2 (22.2)	1 (11.1)	0 (0)	0 (0)	0 (0)	0 (0)	1 (11.1)	0 (0)
**Northwest**	IDU	14	0 (0)	10 (71.4)	0 (0)	4 (28.6)	0 (0)	0 (0)	0 (0)	0 (0)	0 (0)	0 (0)
	Sex	2	1 (50.0)	1 (50.0)	0 (0)	0 (0)	0 (0)	0 (0)	0 (0)	0 (0)	0 (0)	0 (0)
	Blood transfusion	1	0 (0)	0 (0)	1 (100)	0 (0)	0 (0)	0 (0)	0 (0)	0 (0)	0 (0)	0 (0)
	Unknown	20	0 (0)	5 (25.0)	0 (0)	8 (40.0)	4 (20.0)	1 (5.0)	2 (10.0)	0 (0)	0 (0)	0 (0)

IDU, injection drug users.

^a^Data are presented as number (%) of patients.

### Prevalence changes of genotypes 3 and 6a in patients with HIV/HCV co-infection

**Fig 2:**
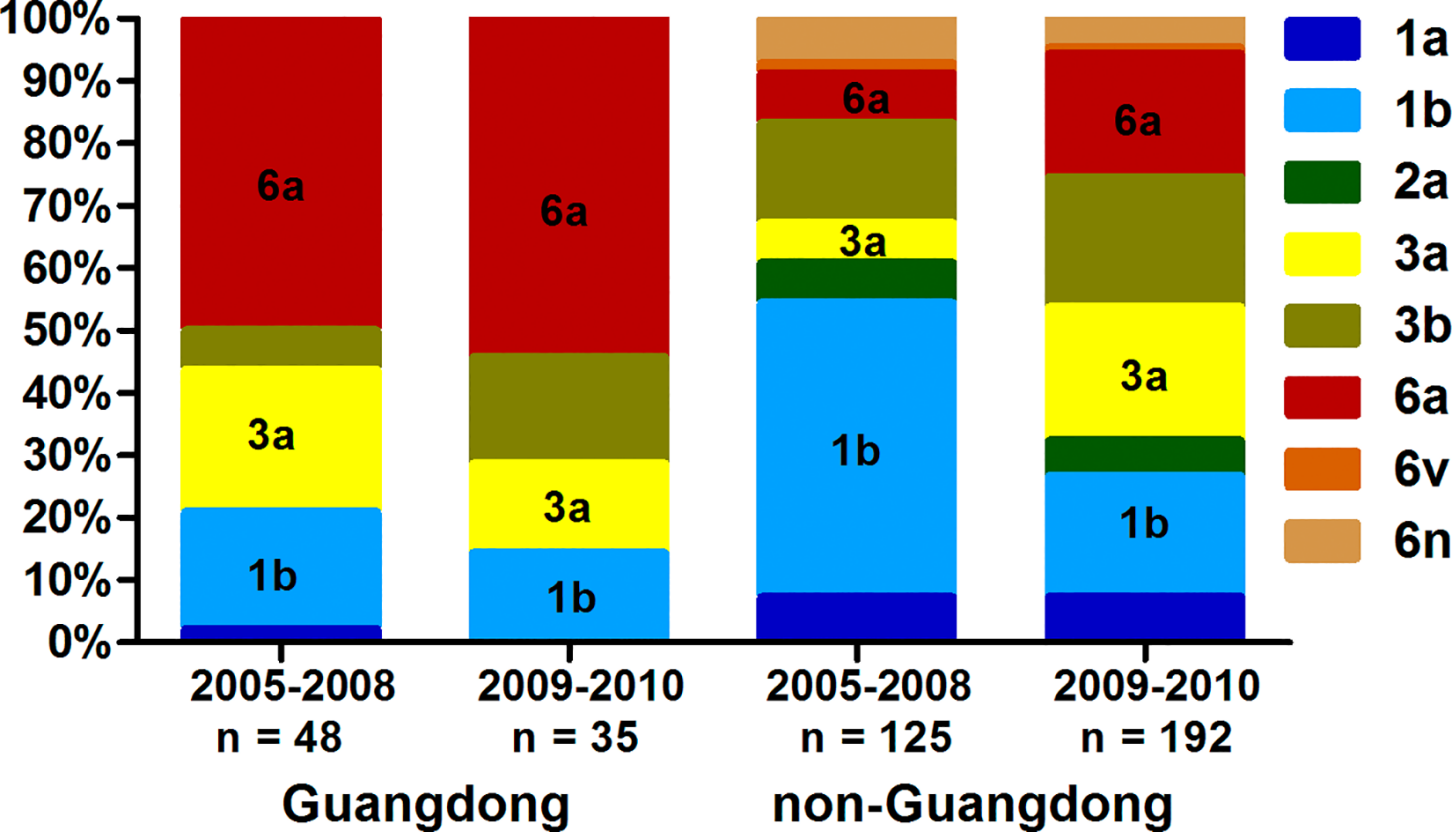
HCV genotype distribution in the Guangdong group and the non-Guangdong group between the 2005–2008 and 2009–2010 cohort. Genotypes 3 and 6a were more common in the non-Guangdong group from 2009–2010 compared to 2005–2008.

A previous study demonstrated the presence of a local epidemic of HCV genotype 6a in Guangdong (province in the South region), which has subsequently led to spread of this genotype to many other regions of China [9]. We sought to verify this data by investigating the prevalence of genotype 6a over time, focusing on geographic regions outside Guangdong. The year of HCV diagnosis, according to the first positive anti-HCV test, was recorded in 400 patients from our study population. To examine temporal shifts in HCV genotypes for HIV/HCV co-infected patients in recent years, we stratified the patients into cohorts by year of HCV diagnosis from years 2005–2008 and 2009–2010; we then further divided patients into a Guangdong group and a non-Guangdong group. In the 2005–2008 and 2009–2010 cohorts, the distribution of genotypes was similar over time in the Guangdong group (P = 0.354), while changes were seen in the non-Guangdong group (P < 0.001). In the non-Guangdong group, genotypes 3 and 6a became more prevalent (22.4% vs. 42.2%, P< 0.001; 8.0% vs. 19.8%, P = 0.004), and the frequency of genotype 1 decreased significantly (54.4% vs. 26.6%, P< 0.001) over time (Fig 2).

## Discussion

A survey from 2006 demonstrated an HCV prevalence rate of 0.43% in the general population of China, but the prevalence of HCV infection in the HIV infected population is estimated to reach from 41.8% up to 87% [10]. The latest epidemiological study on HCV infection in China revealed the largest number of patients were infected with HCV genotype 1b (56.8%), followed by genotypes 2, 3 and 6. The greatest HCV genotypic diversity was observed in the Southern and Western regions of China, which had significantly lower proportions of genotype 1, and correspondingly higher proportions of genotypes 3 and 6 (South) or 2 and 3 (West) [11]. As HCV genotype prevalence can change over time, monitoring these changes can inform clinical management strategies [12]. Although there were many studies describing the HCV genotype distribution, there had rare researches on HCV genotype in HIV/HCV co-infected patients from different regions over time with a large sample size in China. So in this study, we aimed to extend the previous, more limited studies on distribution of HCV genotype in HIV/HCV co-infected patients.

One previous study with a small sample size (n = 20) implied that genotype 6a was the dominant HCV strain in HIV/HCV co-infected patients for IDU in Guangxi [13]. In 2010, Shang et al. explored the HCV genotype distribution in patients with HIV/HCV co-infection, and also found genotype 6 was the major HCV genotype (36.6%) in patients from the South (Yunnan and Guangxi). Conversely, genotype 3 accounted for 65.5% of patients from Xinjiang (Northwest region) while genotype 6 was nearly absent (1/29 patients). Only genotypes 1b and 2a were detected in the Central and Northeast regions (Henan, Liaoning and Jilin) [14]. However, just 218 total patients with HCV genotype results were available in that particular study, the time period (2000–2008) was earlier than our current study, and Guangdong was not included.

In order to provide a better picture of the nationwide distribution of HCV genotype in HIV/HCV co-infection, we enrolled 414 HIV/HCV co-infected patients from three geographically distinct regions, including 9 provinces and cities of China. Our study confirmed prior studies whereby marked differences in the distribution of HCV genotypes varied between geographic regions; in particular, we observed that genotype 6a was the major HCV subtype (30.9%) represented in Southern China, and that the predominant risk factor for transmission was IDU. Almost all patients from the Central region were identified as subtype 1b or 2a (91.7%), and transmission was mainly associated with IDU and contaminated blood transfusion. In contrast, genotype 1 (45.9%) and genotype 3 (43.2%) were equally present in the Northwest region. This may suggest that HCV genotypes 3 and 6 have migrated from South to North in patients with HIV/HCV co-infection when compared to previous studies, as suggested for chronic hepatitis C [7]. In order to determine if HCV genotype was associated with transmission route, we stratified the patients by risk behaviors. Our results indicate that genotypes 3 and 6a account for the majority of IDU from the South (74.8%), while genotypes 1b and 2a were mostly spread through blood transfusion in the South (60%) and exclusively in the Central region. These findings suggest an association between HCV genotype and route of transmission, with increased frequency of genotype 6a in IDU and genotypes 1b and 2a in patients with previous blood transfusion. Other studies in HCV mono-infected patients corroborate these results [7,11]. We also analyzed some patients' HIV genotype. Our results revealed the main HIV strains were subtype 01_AE, 08_BC and 07_BC in the South, subtype 07_BC in the Northwest and subtype B' in the Center region respectively (data not shown). However, the main purpose of this study is to investigate the distribution of HCV genotype, so they were not put into the present study.

Guangdong, located in the South region and bordered by the Guangxi, Hong Kong, Macau and Fujian regions, is an internal and international crossroads for cultural and commercial exchanges, and was the first region to adopt a free market economy and open door policy in China. The continuous socio-economic advancement and migration flow in and out of Guangdong may influence the HCV genotype distribution pattern at both the local and national levels. Moreover, the resulting powerful economy in Guangdong has likewise stimulated a large population of IDU and led to a rapid increase in drug trafficking and trading, resulting in new recombinant HCV strains [9]. Genotype 6a was first found in Guangzhou, the capital city of Guangdong, in 2002 [8] and has become one of the dominant genotypes in this region since 2004 [15]. Two important findings on genotype 6a can be concluded from recent research [9,16]. First, it originated from Vietnam, and has become a local epidemic in Guangdong, subsequently spreading to other regions in China. Second, the frequency of genotype 6a has increased significantly among blood donors in other regions over time. We investigated changes in the distribution of HCV genotypes between 2005 and 2010 in the Guangdong group compared to the non-Guangdong group. Consistent with previous reports [16], there were no obvious changes in the Guangdong group. Outside Guangdong, however, an increasing prevalence of genotypes 3 and 6a and decreasing prevalence of genotype 1 were detected from 2009–2010 compared to pre-2009. Moreover, patients with genotypes 3 and 6a groups are younger on average than the genotype 1b group (Tables 2 and 3). These findings suggest that a further increase in prevalence of genotypes 3 and 6a may occur in the upcoming years, stemming from this younger infected generation. The likely explanation for the decline in genotype 1 is the implementation of mandatory screening for HCV prior to blood donation which started in the 1990s, and led to a dramatic decline in new cases of hepatitis C resulting from contaminated blood transfusion (major HCV subtype 1b). In the meantime, the primary routes of HCV transmission shifted to IDU and sexual contact, which accounted for 56.7% of infections in the Central region in this study. We also postulate that migration from an endemic area to new regions may play an important role. Furthermore, the decreased prevalence of genotype 1 could be beneficial in terms of treatment outcomes.

Treatment with pegylated interferon (PEG-IFN) and ribavirin (RBV) is still the standard of regiment for chronic hepatitis C patients in China. Genotype 1 has been shown to be more refractory to treatment than genotypes 2 and 3 [17,18]. Numerous studies have demonstrated HIV/HCV co-infected patients receiving dual therapy with PEG-IFN/RBV have lower sustained virologic response (SVR) rates compared with HCV mono-infected patients. In a meta-analysis of seven randomized trials in HIV/HCV co-infected patients treated with PEG-IFN/RBV, SVR rates were significantly higher among patients with HCV genotypes 2 or 3 compared to those with genotypes 1 or 4 [19]. These SVR rates are about 10–20% lower than genotype-matched HCV mono-infected patients [20]. A phase II trial, including 60 HIV/HCV co-infected patients, revealed that the SVR rate was 74% among the 38 people in the telaprevir arm, which was similar to the rate in HCV mono-infected patients [21]. Another recent Phase II study was conducted in HIV/HCV co-infected patients (genotype 1 or 4) receiving 24 weeks of asunaprevir, daclatasvir and PEG-IFN/RBV. The global SVR12 rate was 96.0% overall, 92.6% in cirrhotic patients, 94.6% in genotype 1 and 97.4% in genotype 4 patients [22]. However, few clinical trials and no standard therapeutic regimen have been described for HIV/HCV co-infected patients with HCV genotype 6 so far.

Although a large number of patients were included in the present study, several limitations exist. First, we did not recruit any patients from the Northeast region of China, and the number of patients recruited from the Central and Northwest regions were relatively small. Previous research has shown only genotypes 1b and 2a are present in the Northeast (Liaoning and Jilin). Future studies should span nationwide, thereby encompassing more regions and including a larger sample size. Second, this was a retrospective, cross-sectional and hospital-based study. The optimal way to confirm our results would be to perform a longitudinal cohort study with patients recruited from the general population. We do believe, however, that our results reflect the current distribution of HCV genotype in the HIV/HCV co-infected population in China.

In conclusion, this study reveals the changing epidemiology of HCV genotype among patients with HIV/HCV co-infection in China. The dominant frequency of HCV genotypes 3 and 6a in the South, as well as their regional spread and increased prevalence outside Guangdong, is concerning with IDU as an ongoing route of transmission. These findings may have important implications for HIV/HCV transmission and treatment in China.
